# The Effect of Acid-Reducing Pharmacotherapy on the Severity of Nausea and Vomiting of Pregnancy

**DOI:** 10.1155/2009/585269

**Published:** 2009-07-01

**Authors:** Simerpal Kaur Gill, Caroline Maltepe, Katayoon Mastali, Gideon Koren

**Affiliations:** ^1^The Motherisk Program, The Hospital for Sick Children, 555 University Avenue, Toronto, ON, Canada M5G 1X8; ^2^Department of Pharmacology, University of Toronto, Toronto, ON, Canada M5S 1A8

## Abstract

*Background*. Heartburn and acid reflux (HB/RF) are associated with increased severity of nausea and vomiting. The ability of acid-reducing drugs to reduce symptoms of nausea and vomiting of pregnancy has not been previously tested. *Objective*. To determine whether acid-reducing pharmacotherapy decreases the severity of NVP symptoms. *Methods*. We studied a cohort of women experiencing NVP, who were also experiencing HB/RF. Women were counseled to commence acid-reducing pharmacotherapy. The effectiveness of the acid-reducing medication in decreasing symptoms of both HB/RF and NVP was measured. *Results*. Acid-reducing drugs resulted in significant decreases in PUQE (9.6 ± 3.0 to 6.5 ± 2.5, *P* < .0001) and well-being scores from the initial (4.0 ± 2.0) to the follow-up interview (6.8 ± 1.6, *P* < .0001). After intervention with acid-reducing pharmacotherapy, a reduction in acid symptoms correlated significantly with reduction in NVP (*R* ^2^ = 0.72, *P* < .001). *Conclusion*. This is the first study to demonstrate that management of HB/RF can reduce the severity of NVP.

## 1. Introduction

Nausea and vomiting of pregnancy (NVP) is the most common medical condition in pregnancy, experienced by up to 80% of women [1]. NVP has been shown to severely affect a woman's quality of life and her ability to function, especially when improperly managed [2]. Antiemetics are usually successful in managing NVP; however, certain medical conditions or symptoms, such as heartburn and/or acid reflux (HB/RF), can exacerbate the severity of NVP [3].

Heartburn and/or acid reflux are common medical disorders; it has been estimated that the incidence of gastroesophageal reflux disorders in pregnancy ranges between 40% and 85% [4, 5]. Symptoms associated with gastroesophageal reflux disorders or dyspepsia may include heartburn, acid reflux, regurgitation, eructation, flatulence, stomach bloating, indigestion, and sensation of a lump in the throat and may even affect quality of sleep [6, 7]. These aforementioned symptoms can occur any time during pregnancy, and the severity of symptoms ranges possibly as a result of gastrointestinal tract motility changes due to increased levels of circulating sex hormones [8]. Similar changes in gastric motility and dysrhythmias have been observed in women suffering from NVP [8].

In a recent prospective, cohort study, we demonstrated that women experiencing both NVP and heartburn and/or acid reflux (*n* = 194) experienced greater severity of NVP compared to women who did not have any heartburn and/or acid reflux (*N* = 188) after controlling for certain confounders [9]. Trends were observed with more women experiencing both heartburn and acid reflux classifying their NVP as severe compared to controls and compared to women only experiencing either heartburn or acid reflux [9]. However, presently no study has examined the effectiveness of acid-reducing pharmacotherapy on NVP symptoms.

The objective of this study was to quantify whether acid-reducing pharmacotherapy is effective in decreasing the severity of NVP in women experiencing HB/RF.

## 2. Methods

The Motherisk Program, located at the Hospital for Sick Children in Toronto, has a specialized helpline for the management of NVP. Women from Canada and the US experiencing NVP can call a toll-free service (1-800-436-8477) to receive pharmacological and nonpharmacological advice on the management of NVP. This evidence-based counseling is based on research and continuous systematic review of emerging clinical and experimental evidence [10]. 

For the purpose of the present study, we enrolled women counseled by the NVP Helpline from November, 2007 to June, 2008. The study group consisted of all women who experienced heartburn and/or acid reflux while suffering from NVP. As per our standard, evidence-based counseling [10], these women were advised by us to commence on acid-reducing pharmacotherapy, and based on the severity of their HB/RF symptoms and on previous pregnancy use, if any, antacids, histamine 2 blockers, or proton pump inhibitors were recommended. Additionally, as histamine 2 blockers are available over-the-counter in Canada, usually they are recommended initially. All women agreed to continue their antiemetic at the dose taken prior to adding the acid-reducing medication. Women who changed their antiemetic dose were excluded from analysis. 

A standard interview was conducted, where detailed quantification of symptoms was obtained using the following validated tools: (1) the Pregnancy-Unique Quantification of Emesis and nausea (PUQE) score [11] (Table 1); (2) the well-being score [12] ranging from 0–10 was recorded based on how the woman felt overall compared to how she felt before pregnancy; (3) a self-report of how the woman perceived her symptoms (mild, moderate, severe). In addition, we recorded the time of onset of the NVP symptoms, gravidity, maternal age at conception, gestational age at the initial interview, and at follow-up, medical conditions that are associated with increased severity of NVP, medication use and the severity of NVP in previous pregnancies.

A standard follow-up interview was subsequently conducted to determine PUQE and Well-being scores and to inquire as to the acid-reducing pharmacotherapy used. To determine the role of acid-reducing pharmacotherapy in decreasing the severity of NVP, women were asked to rate on a scale of 0–10, the effectiveness of their medication in reducing their acid symptoms, and the effectiveness of this medication in reducing their NVP.

Paired *t*-test was used to compare the mean PUQE and Well-being scores between the initial and follow-up interviews. Linear regression was used to determine the relationship between the reduction in heartburn and acid reflux and NVP. Similarly, linear regression was also performed on the initial PUQE scores and the change in PUQE scores, and on the onset of NVP and the onset of symptoms of HB/RF.

Statistical analyses were conducted with the SigmaStat program version 3.1 (Systat Software Inc., 2000, Ill, USA).

## 3. Results

Of 140 women, there were 80 women who experienced HB/RF but were not stabilized on antiemetics and therefore were excluded from our analysis. The final cohort consisted of 60 women with NVP: 14 experienced only heartburn, 35 experienced only acid reflux, and 11 reported on both heartburn and acid reflux. Of the women included in our analysis, the self-reported severity of NVP was as follows: 72% of women classified their NVP as severe, 19% as moderate, and 9% as mild.

Mean gestational ages at initial counseling and at follow-up were 9.6 ± 3.8 weeks and 12.4 ± 2.1 weeks, respectively. Mean gestational age at onsets of NVP was at 5.5 ± 3.0 weeks, and mean gestational age at which symptoms of HB/RF occurred was 6.8 ± 2.4 weeks. Additionally, linear regression demonstrated that the onsets of NVP and HB/RF were significantly correlated (*R* ^2^ = 0.25, *P* = .004).

**Figure 1:**
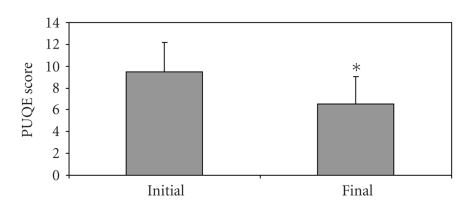
PUQE scores of women experiencing HB/RF and NVP at initial call and at follow-up after the use of acid-reducing pharmacotherapy; Final (marked “∗”): *P* < .0001, compared to control.

**Figure 2:**
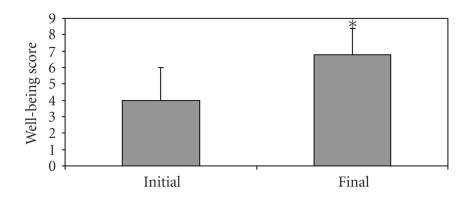
Well-being scores of women experiencing HB/RF and NVP at initial call and at follow-up after the use of acid-reducing pharmacotherapy; Final (marked “∗”): *P* < .0001, compared to control.

There were no significant differences in PUQE scores of women excluded from the study and initial PUQE scores of women included in the study (9.5 ± 2.5 and 9.6 ± 3.0, *P* = .2376). Use of acid-reducing medication resulted in a significant decrease in PUQE scores at follow-up (from 9.6 ± 3.0 to 6.5 ± 2.5, *P* < .0001, Figure 1). Similarly, there was a significant improvement in the Well-being scores from the initial (4.0 ± 2.0) to the follow-up interview (6.8 ± 1.6, *P* < .0001, Figure 2).

**Figure 3:**
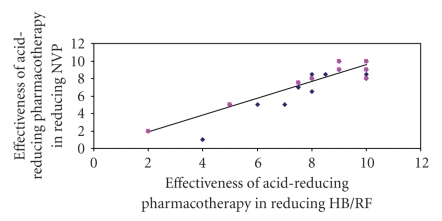
Linear regression comparing the effectiveness of acid-reducing pharmacotherapy in reducing HB/RF, and in reducing NVP. Women rated effectiveness from zero (no effect) to 10 (maximal effect); *R* ^2^ = 0.72, *P* < .001.

The most commonly used acid-reducing pharmacotherapy was histamine-2 blockers, used by two-thirds of women (40/60). Proton pump inhibitors were used by 13 out of 60 women, and other over-the-counter antacids were used by 7 out of 60 women. The mean effectiveness of acid-reducing pharmacotherapy rated by the women was 8.2 out of 10, and the mean effectiveness of the acid-reducing pharmacotherapy in reducing NVP was 7.7 out of 10. Women noticed improvement, on average, 3-4 days after commencing acid-reducing pharmacotherapy. Linear regression demonstrated that a reduction in acid symptoms significantly predicted the reduction in NVP with the use of acid-reducing pharmacotherapy (*R* ^2^ = 0.72, *P* < .001, Figure 3). As the severity of PUQE increased, there was a greater reduction in PUQE scores after the use of acid-reducing pharmacotherapy as demonstrated by linear regression (*R* ^2^ = 0.15, *P* = .003).

## 4. Discussion

Our data demonstrate for the first time that acid-reducing pharmacotherapy reduces the severity of NVP. There was a strong correlation between the reduction in acid symptoms and the reduction in the severity of NVP suggesting that treatment of HB/RF will cause improvement in NVP. Women reported an improvement in both HB/RF and NVP symptoms within 3 to 4 days after starting acid-reducing pharmacotherapy. Furthermore, women experiencing the most severe NVP had the greatest change in their NVP after using acid-reducing pharmacotherapy. These results support our initial observational study [9] in suggesting that HB/RF is a significant contributor to NVP. Additionally, the onset of symptoms of NVP significantly correlated with the onset of symptoms of HB/RF providing further evidence that HB/RF exacerbates NVP.

Since withholding treatment was not considered ethical in the context of our clinical practice in the NVP healthline, our study could not recruit a cohort of women experiencing symptoms of HB/RF who did not use acid-reducing pharmacotherapy. The lack of a comparison group is a limitation; however, the results from this pilot study provide valuable data for a future controlled study. To ensure, however, that the potential effect of acid-reducing drugs on NVP severity can be attributed to these medications, we excluded women who increased their antiemetics during the study. This exclusion was done prior to evaluating the potential effects of acid suppressing drugs on the severity of NVP.

Nausea and vomiting of pregnancy and HB/RF result in adverse maternal outcomes including decreasing a woman's quality of life and her ability to function [1, 2], and more serious gastrointestinal morbidities such as gastroesophageal reflux disorder or peptic ulcers [13]. Treatment of HB/RF by histamine 2 blockers or proton pump inhibitors should be considered to alleviate symptoms, especially since these classes of drugs have been quite well studied in pregnancy, and have not been associated with increased fetal risks [14–28].

## Figures and Tables

**Table 1:** Motherisk-pregnancy-unique quantification of emesis and nausea (PUQE) scoring system. The PUQE scale is a validated scoring system to quantify the severity of NVP based on quantification of the 3 physical symptoms of NVP (nausea, vomiting, and retching) [11].

How many hours in past 24 hours had you felt nauseated/sick to stomach?	None ( 1)	1 hour or less ( 2)	2-3 hours ( 3)	4–6 hours ( 4)	>6 hours ( 5)
How many times in the past 24 hours did you vomit?	≥7 times ( 5)	5-6 times ( 4)	3-4 times ( 3)	1-2 times ( 2)	None ( 1)

How many times in the past 24 hours did you experience gagging or retching or dry heaves?	None ( 1)	1-2 times ( 2)	3-4 times ( 3)	5-6 times ( 4)	≥7 times ( 5)
